# Phylogenetic and morphological influence on habitat choice in moisture‐harvesting horned lizards (*Phrynosoma* spp.)

**DOI:** 10.1002/ece3.8132

**Published:** 2021-09-21

**Authors:** Anna‐Christin Joel, Jenice R. N. Linde, Philipp Comanns, Caroline Emonts, Margret Weissbach, Morris Flecks, Dennis Rödder

**Affiliations:** ^1^ Institute of Biology II RWTH Aachen University Aachen Germany; ^2^ Zoologisches Forschungsmuseum Alexander Koenig Bonn Germany

**Keywords:** adaptation, desert, drinking, rain harvesting, skin, wettability

## Abstract

In previous studies, the superhydrophilic skin of moisture‐harvesting lizards has been linked to the morphological traits of the lizards’ integument, that is, the occurrence of honeycomb‐shaped microstructures. Interestingly, these structures can also cover the skin of lizards inhabiting wet habitats. We therefore tested the influence of the microstructures’ main features on the habitat choice and wettability in the genus *Phrynosoma*. The genus *Phrynosoma* comprises moisture‐harvesting species as well as nonspecialists. Lizards of this genus inhabit large areas of North America with diverse climatic conditions. Remarkably, the differences in the manifestation of microstructures are just as versatile as their surroundings. The phylogeny of the lizards as well as the depth of their ventral microstructures, though independent of each other, correlated with the precipitation in their respective habitat. All other morphological traits, as well as the skin's wettability itself, could not predict the habitat of *Phrynosoma* species. Hence, it is unlikely that the microstructure influences the wettability, at least directly. Hence, we presume an indirect influence for the following reasons: (a) As the ventral side cannot get wet by rain, but the belly could easily interact with a wet surface, the microstructure might facilitate water absorption from wet soil following precipitation. (b) We found the number of dorsal microstructures to be linked to the occurrence of silt in the habitat. In our study, we observed scales being heavily contaminated, most likely with a mixture of dead skin (after shedding) and silt. As many lizards burrow themselves or even shovel sand onto their backs, deploying the substrate might be a mechanism to increase the skin's wettability.

## Graphical abstract

Lizards inhabiting arid regions have to harvest water from various sources, as water is scarce. These moisture‐harvesting lizards are suspected to have a specialized microstructure on their skin, rendering the skin superhydrophilic. However, our data do not support this hypothesis. We found soil particles accumulating in the structures, implying that microstructures could function as some sort of "container", accumulating soil and dirt and thus changing the surface chemistry.

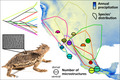

## 1 INTRODUCTION

High temperatures as well as rare and irregular precipitation characterize arid regions across the world. To survive in such a harsh environment, animals require specialized adaptations, for example, avoiding of overheating and gathering enough water. Efficient water collection from rain, moist air, or soil has been described as “moisture harvesting” and evolved independently in several species (Cardwell, 2006; Lillywhite & Stein, 1987; Norgaard & Dacke, 2010; Repp & Schuett, 2008; Sherbrooke et al., 2007; Yenmiş et al., 2015). Moisture‐harvesting lizards, as one example for these species, show a stereotypical behavior: In several species of the genera *Phrynocephalus*, *Trapelus*, and *Phrynosoma*, this behavior includes (a) lowering of head and tail, (b) raising of abdomen in an arch, (c) splaying of legs, and (d) flattening of the body (Schwenk & Greene, 1987; Sherbrooke, 1990, 2004; Vesely & Modry, 2002). On the other hand, the integument (skin and appendages like scales) of such lizards shows specific adaptations as well. Whereas in wet habitats, like rainforests, the lizards’ scales exhibit superhydrophobic and self‐cleaning properties to prevent infections (Joel & Weissbach, 2019; Watson et al., 2015), in arid regions they have to prevent water evaporation from their body and simultaneously absorb, retain, and/or facilitate drinking of water (Comanns, 2018; Joel et al., 2018).

The reptilian epidermis in general consists of an inner α‐ and an outer β‐layer, mainly composed of α‐ and β‐keratins, respectively. These two layers are divided by the mesos layer (Alibardi & Maderson, 2003; Maderson et al., 1998). Because of its composition of lipids, it is responsible for the water impermeability of the lizard's integument and hence protects the lizard from desiccation. The outer β‐layer is covered by the corneous protective Oberhäutchen (Nick, 2014). For harvesting water, the lizards’ scales overlap, forming an interconnected channel network (Comanns et al., 2015; Gans et al., 1982; Sherbrooke et al., 2007). Because of capillary forces within these channels, water transportation takes place passively and sometimes even with a pre‐determined flow direction toward the mouth (Comanns et al., 2011, 2015, 2016; Yenmiş et al., 2015). In addition to this channel network, the lizards must have a hydrophilic integument. The hydrophilicity was so far linked to a honeycomb‐shaped microstructure (or microornamentation) on the Oberhäutchen, keeping a film of water after pre‐wetting (Berthé et al., 2009; Comanns et al., 2011; Hofling & Renous, 2009; Riedel et al., 2015; Sherbrooke et al., 2007; Vesely & Modry, 2002; Yenmiş et al., 2015). This microstructure, however, is also found on nonmoisture‐harvesting reptiles, such as the modest forest dragon *Hypsilurus modestus* (own observation) or the smooth helmeted iguana *Corytophanes cristatus* (Lang, 1989).

Our study focuses on the genus *Phrynosoma* (horned lizards), which comprises at least three moisture‐harvesting species (*P. cornutum*, *P. modestum*, and *P*. *platyrhinos*) as well as at least one nonspecialist (*P. hernandesi*) (Dodge, 1938; Peterson, 1998; Sherbrooke, 1990, 2002). Additionally, members are endemic to southwestern regions of North America and inhabit mainly, but not only, arid habitats (Baur & Montanucci, 1998; Bryson et al., 2012; Lara‐Resendiz et al., 2014, 2015; Newbold & MacMahon, 2014; Sherbrooke, 2002; Zamudio & Parra‐Olea, 2000). In case the microstructure does indeed entail increased wettability, we assume that *Phrynosoma* species inhabiting drier areas exhibit more and/or deeper honeycomb‐shaped structures on their Oberhäutchen. Additionally, we assume that closely related species would inhabit similar habitats, as their morphological features would be beneficial for colonization. Hence, in this study we analyze the linkage between microstructure, phylogeny, habitat choice as well as wettability of *Phrynosoma* spp.

## 2 MATERIAL AND METHODS

### 2.1 Study animals

Six captive‐bred *Phrynosoma platyrhinos* were kept in an arid terrarium (146 cm × 55 cm × 80 cm). A soil mixture consisting of sand and clay was used as substrate. Below the heat lamp, temperature was 45–50°C, whereas the cooler regions of the terrarium were 28°C. The lizards were fed with ants, cowpea weevils, and microcrickets. Please refer to Baur and Montanucci (1998) for further information about keeping *Phrynosoma*.

Additional to samples of our individuals, shed skin of captive‐bred *P. asio*, *P. cerroense*, *P. coronatum*, *P. goodei*, *P. hernandesi*, *P. orbiculare*, *P. modestum,* and *P. taurus* was kindly provided by private breeders. Please note that we were not able to match these samples to individuals within one species, and thus, there might be pseudo‐replicates included in the shed skin data.

Museum specimens of wild‐caught *P. asio*, *P. cerroense*, *P. cornutum*, *P. hernandesi*, *P. orbiculare*, *P. platyrhinos*, *P. solare*, *P. taurus*, and *Moloch horridus* were obtained from the collection of the Zoological Research Museum Alexander Koenig, Bonn, Germany. The used biological material is also listed in the supplement (Table S1).

### 2.2 Wetting experiments

We refrained from using museum specimens for wettability tests, as we observed ruptures of the Oberhäutchen in several specimens, leading to an absorption of the applied fluid by the subjacent skin layers. Hence, we chose to use only shed skin for wetting experiments. Shed skin samples exhibiting a rupture within the region of interest were discarded.

Three pieces of shed skin, ventrally and dorsally, were taken for examination. Scale structure differs between ventral and dorsal sides in Phrynosomatidae, making discrimination easy. The pieces of shed skin were placed onto a glass slide, and 2 µl of distilled water with 0.6% blue food dye (Queen Fine Foods PTY Ltd., Alderley, Australia) for coloration was applied. Spreading of the liquid was observed via a high‐speed recording microscope (250 fps, VW‐9000C; Keyence Cooperation, Osaka, Japan). The differences (%) of the area covered by the droplet (0 to 60 s) were evaluated with the help of a customized Python (3.5) script. For calculation of mean and statistics, we replaced data for perfect wetting (i.e., droplet wets complete skin within 60 s and thus a rim is unidentifiable) with the largest value we were still able to measure in our data set (raw data: Table S7).

We validated our data with the wettability of living animals (*P. asio*, *P. modestum*, *P. platyrhinos*, *P. solare*, *P. taurus*). For this, a droplet of tap water was applied to the dorsal side of the animal to visually check whether spreading occurs (“yes” vs. “no”). For all but *P. platyrhinos*, these data were provided by private breeders.

### 2.3 Cleaning of samples

All skin samples were contaminated to varying degrees. This was not only true for museum specimens but also for shed skin samples. Hence, the contamination is not (only) due to storage and/or age of the museum samples. Different cleaning methods were exploited to remove the contamination on the shed skin of *P. platyrhinos*, without any success:

We first used an ultrasonic bath with different cleaning agents (distilled water, 70% ethanol, and chloroform) for 30 s and 60 s. In a second approach, shed skin samples were incubated and swirled for 90 min in chloroform and subsequently treated for 30 s in chloroform in the ultrasonic bath. The same treatment was carried out with soapy water and the final cleaning with distilled water. Alternatively, the shed skin pieces were either fixed with double‐sided sticky tape and cleaned with a fine brush and/or compressed air of 10 bar, or a second stripe of adhesive tape was slightly pressed onto the distal side of the shed skin and then slowly pulled off. In a last trial, the shed skin was fixed upside down to a centrifugation tube, either empty or filled with distilled water. The samples were then centrifuged with 2,000 g for 2 min.

### 2.4 Morphological examination of microstructures

Skin samples (approx. 5 × 10 mm, ventrally and dorsally) were taken with a scalpel from preserved museum specimens of *P. asio*, *P. cerroense*, *P. cornutum*, *P. hernandesi*, *P. orbiculare*, *P. platyrhinos*, *P. solare*, and *P*. *taurus*. Samples were dehydrated by an ascending ethanol series. Remaining alcohol was washed out with hexamethyldisilazane and dried overnight. Afterward, one side of the sample was clean cut with a razor blade. Slices were either placed flat or with the cutting edge facing up on SEM plates. Afterward, samples were sputtered with gold and observed with a scanning electron microscope (Philips AG, Amsterdam, the Netherlands). Shed skin samples of *P. asio*, *P. cerroense*, *P. coronatum*, *P*. *goodei*, *P. hernandesi*, *P*. *modestum*, *P. orbiculare*, *P. platyrhinos,* and *P*. *taurus* were prepared as described above without the dehydration step.

Data are presented as mean ± *SD*, where *SD* is the standard deviation (raw data: Table S7, including biological material source). SEM samples were analyzed using Keyence VW‐9000 Software (version 1.4.0.0). The clean cut samples were used to measure the depth of the microstructure (with n as high as possible for each sample), whereas the flat samples were used to calculate the microstructures per mm^2^ (with three spots per sample).

### 2.5 Histology

Histological examinations were performed with skin samples of museum specimens of *P. solare*, *P. cornutum*, and *M. horridus*. As museum specimens, they were wild‐caught animals and not bred for several generations on a non‐natural substrate. Thus, the contamination should appropriately reflect any contamination occurring in the wild. Samples were prepared and dehydrated as described before and then cut with razor blades into slices of ca. 1 mm width. These slices were embedded in epoxy resin. Semi‐thin sections of ~0.7 µm thickness were cut with an OM U3 microtome (Reichert, Vienna, Austria). For the subsequent staining, the epoxy resin was removed as described by Mayor et al. (1961). Prepared sections of each lizard and body side were stained with either methylene blue staining, alizarin red staining, periodic acid‐Schiff staining, or gram staining (Mulisch & Welsch, 2015). Cover‐slips with sections, stained with methylene blue, alizarin red, or periodic acid‐Schiff stain, were glued to an object slide using Merckoglas. For gram‐stained sections, Canada balsam was used for final fixation. All sections were examined with a BA310 digital microscope (Motic Deutschland GmbH, Wetzlar, Germany).

### 2.6 Phylogenetic tree

Depending on the taxonomic concept, the genus *Phrynosoma* currently comprises 17–21 species (Montanucci, 2015; Uetz et al., 2020; Zamudio et al., 1997). However, by testing several species delimitation methods, Blair and Bryson (2017) found no support for the classification proposed by Montanucci (2015), who resurrected the names *P. brevirostris* and *P. ornatissimum* and used morphological information to describe two new taxa (*P. bauri* and *P. diminutum*). Therefore, we follow the taxonomy proposed by Blair and Bryson (2017) within the present study.

Phylogenetic relationships within *Phrynosoma* were inferred with BEAST version 1.8.3 (Drummond et al., 2012), which estimates phylogeny using Bayesian MCMC. We used previously published data (Hodges & Zamudio, 2004; Leaché & McGuire, 2006; Nieto‐Montes de Oca et al., 2014) of six nuclear loci (BDNF, EXPH5, NKTR, R35, RAG1, and SOCS5, Tables S2 and S3). Substitution models were calculated using ModelTest (Posada & Crandall, 1998) as implemented in the package phangorn for Cran R. Model parameters were estimated separately for each locus, but loci were not partitioned by codon positions in order to avoid overparameterization that impeded the MCMC mixing. The Yule speciation process was set as tree prior to using a random starting tree. Results of the BEAST analyses were summarized from four independent runs with 2 × 10^7^ generations each, sampling every 2 × 10^3^ trees and omitting the initial 10% as burn‐in after checking for convergence and sufficient effective sample sizes with Tracer v1.7 (Rambaut et al., 2018).

Divergence dates were estimated via a relaxed molecular clock approach with an estimated rate and uncorrelated lognormal distribution (Drummond et al., 2006). Three fossil taxa were used for calibration: *Paraphrynosoma greeni* (33.3 mya, stem of *Phrynosoma*; Holman, 1987; Smith, 2006; Wiens et al., 2013), *Phrynosoma* sp. (13.6 mya, oldest known fossil from the *P. douglasii* group; Estes & Tihen, 1964; Bryson et al., 2012), and *Phrynosoma anzaense* (1.8 mya, sister of *P. mcallii*; Norell, 1989) (Table S4). Fossils represent minimum ages of clades; thus, they were implemented as single‐sided hard bounds by using a diffuse gamma shaped prior distribution with the fossil's age as offset.

### 2.7 Hydrological and pedological niche assessment

With a spatial resolution of 30 arc seconds, climatic information characterizing humidity and precipitation conditions within the range of the studied *Phrynosoma* species was obtained from the CHELSA database representing an average between 1979 and 2013 (Karger et al., 2017). As we were predominantly interested in the hydric niche of the species, we downloaded monthly mean, minimum, and maximum temperature as well as precipitation and computed bioclimatic variables as listed in Table 1 using the ENVIREM package for R (Title & Bemmeis, 2017). These variables describe annual averages, extremes, and different aspects of the conditions the species experience within their habitats. Therefore, these parameters are likely to represent the selective landscape influencing the morphological adaptations associated with moisture harvesting. Soil data for the average conditions within a depth range of 0–5 cm and with a spatial resolution of 250 m were obtained from the SoilGrids database (https://www.isric.org/explore/soilgrids).

**TABLE 1:** Definitions and abbreviations of bioclimatic variables. A: Zomer et al. (2008); B: Hargreaves et al. (1985); C: Thornthwaite (1948); D: Busby (1991) E: Willmott and Feddema (1992); J: Vörösmarty et al. (2005); K: Metzger et al. (2013); L: www.soilgrids.org vers. 2.0., average conditions at a depth range of 0–5 cm. PET = potential evapotranspiration

Abbreviation	Description	Units	Source
annualPET	Annual potential evapotranspiration: a measure of the ability of the atmosphere to remove water through evapotranspiration processes, given unlimited moisture	mm/yr	A, B
aridityIndexThornthwaite	Thornthwaite aridity index: index of the degree of water deficit below water need	–	C
bio_12	Annual Precipitation	mm/year	D
bio_13	Precipitation of Wettest Month	mm/month	D
bio_14	Precipitation of Driest Month	mm/month	D
bio_15	Precipitation Seasonality	Coefficient of variation	D
bio_16	Precipitation of Wettest Quarter	mm/quarter	D
bio_17	Precipitation of Driest Quarter	mm/quarter	D
bio_18	Precipitation of Warmest Quarter	mm/quarter	D
bio_19	Precipitation of Coldest Quarter	mm/quarter	D
climaticMoistureIndex	A metric of relative wetness and aridity	–	E, J
PETColdestQuarter	Mean monthly PET of coldest quarter	mm/month	K
PETDriestQuarter	Mean monthly PET of driest quarter	mm/month	K
PETseasonality	Monthly variability in potential evapotranspiration	mm/month	K
PETWarmestQuarter	Mean monthly PET of warmest quarter	mm/month	K
PETWettestQuarter	Mean monthly PET of wettest quarter	mm/month	K
cfvo	Volumetric fraction of coarse fragments (>2 mm)	cm^3^/dm^3^ (vol‰)	L
clay	Proportion of clay particles (<0.002 mm) in the fine earth fraction	g/kg	L
sand	Proportion of sand particles (>0.05 mm) in the fine earth fraction	g/kg	L
silt	Proportion of silt particles (≥0.002 mm and ≤0.05 mm) in the fine earth fraction	g/kg	L
soc	Soil organic carbon content in the fine earth fraction	dg/kg	L

As geographic range surrogates, georeferenced records for each species were obtained from the global biodiversity information facility (www.gbif.org) and the VertNet database (www.vertnet.org), wherein the final data set comprised in total 8,844 unique records (115 of *P. asio*, 1,143 of *P. blainvillii*, 44 of *P. braconnieri*, 191 of *P. cerroense*, 2,589 of *P. cornutum*, 2,183 of *P. coronatum*, 9 of *P. ditmarsi*, 40 of *P. douglasii*, 105 of *P. goodei*, 699 of *P. hernandesi*, 314 of *P. mcallii*, 873 of *P. modestum*, 554 of *P. orbiculare*, 1,574 of *P. platyrhinos*, 6 of *P. sherbrookei*, 407 of *P. solare*, and 74 of *P. taurus*).

Phylogenetic signals in hydric and pedological niche dimensions and morphological traits were assessed using two approaches: Pagel's lambda (Pagel, 1999) and Blomberg´s K (Blomberg et al., 2003) using the phylosignal package for R (Keck et al., 2016).

Based on each set of records, the median position of the species in climate space was computed and median traits of all three morphological variables were tested in a phylogenetic general least‐squares (PGLS) framework in R using a Brownian motion model as phylogenetic constrain (Orme et al., 2018). We acknowledge the possibility of alpha error accumulation using false discovery rate (fdr) adjustment (p.adjust function in R). We performed a principal component analysis using 10,000 random samples throughout the study area considering those variables, where we detected a significant relationship between morphological features and environmental parameters irrespective of potential alpha errors (see results). Based on the median positions of each species in the resulting principal components, we reconstructed ancestral conditions using the “fastBM” function and trait diagrams using the “phenogram” function of the phytools package for R (Revell, 2012). These trait diagrams illustrate a projection of the phylogenetic tree in a space defined by the climatic niche and time (Evans et al., 2009). The niche breadth of each species was illustrated using density profiles (function “ggdensity,” “ggpubr,” and “ggplot2” packages for R; Wickham (2016) and Kassambra (2020)). The multidimensional niche position and volume were quantified by computing hypervolumes based on support vector machines (hypervolume package for R; Blonder, 2015; Blonder et al., 2014).

## 3 RESULTS

### 3.1 Phylogenetic tree and divergence times

**FIGURE 1:**
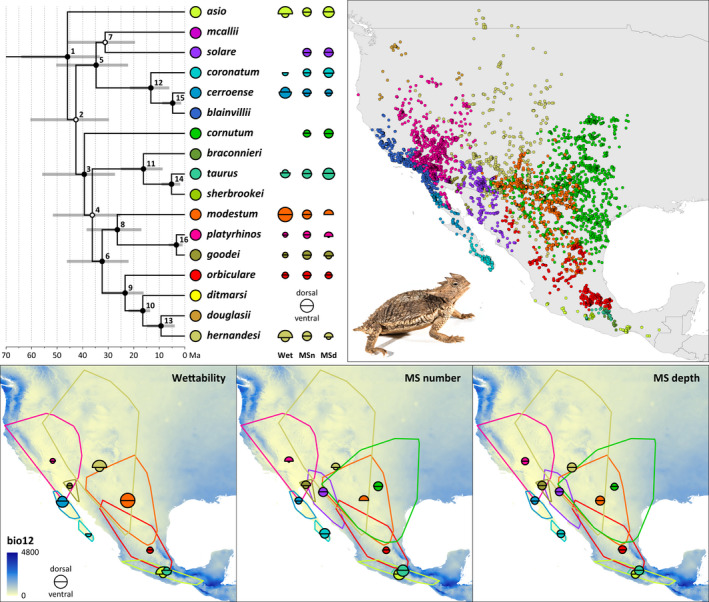
Dated phylogeny of the genus *Phrynosoma* (left), traits measured (middle) as well as distribution records used in this study (right). The tree was obtained from a Bayesian relaxed‐clock analysis of six nuclear loci. The outgroup (*Sceloporus bicanthalis*) is not shown for clarity. Gray bars show 95% highest posterior density (HPD) of divergence times, numbers on nodes refer to Table S4, where the detailed values are given. Solid nodes have posterior probabilities >.95 and the topology presented here is identical to the fully supported phylogeny based on phylogenomic data of Leaché and Linkem (2015). Next to the phylogenetic tree are the mean values for wettability (wet) as well as microstructure number (MSn) and depth (MSd), dorsally and ventrally of each species tested (compare to Tables S5 and S6). These traits are also presented below linked to habitat and annual precipitation (bio12). The traits are placed as centroid within the convex hull of the distribution records for each species. Inset: *Phrynosoma taurus*

Our dated phylogeny of *Phrynosoma* obtained from the BEAST analyses is generally well supported, with most nodes having posterior probabilities >.95 (Figure 1). The inferred relationships largely reflect the subgeneric divisions of *Phrynosoma* and the topology is in concordance to previously published phylogenies of the genus (Leaché & Linkem, 2015; Leaché & McGuire, 2006; Wiens et al., 2013). Estimated divergence times (Table S4) between clades or species range between 45.93 mya (95% highest posterior density intervals 33.30–64.00 mya) and 3.33 mya (0.87–6.28 mya).

### 3.2 Phylogenetic signal in hydric niche dimensions

Based on phylogeny, we analyzed the dispersion of *Phrynosoma* across America. We indeed found strong evidence that the habitat choice of *Phrynosoma* is in dependence on its phylogenetic origin. There were significant phylogenetic signals analyzing hydric as well as the pedological niche dimensions (Table 2; i.e., Thornthwaite aridity index, annual mean precipitation (bio_12), minimum (bio_14) and maximum (bio_13) precipitation of the driest/wettest month, precipitation of the wettest quarter (bio_16), the climatic moisture index, and the proportion of soil organic carbon compounds (soc) and clay). As suggested by both Pagel's lambda and Blomberg's *K*, a Brownian motion model of evolution may explain best the relationships in hydric niche dimensions. Phylogenetic signals in pedological niche dimensions were less pronounced.

**TABLE 2:** Significant phylogenetic signals in terms of Pagel's lambda and Blombergs *K* and K* in hydric and pedological niche dimensions (*p* < .05). For abbreviations, see Table 1. Appendix 1 comprises data of all analyses

Variable	Phylogenetic signal			Significance (*p*‐value)		
	*K*	K*	Lambda	*K*	K*	Lambda
aridityIndexThornthwaite	1.16	1.15	1.05	0.02	0.02	0.05
bio_12	1.30	1.22	1.06	0.01	0.02	0.03
bio_13	1.23	1.14	1.08	0.02	0.03	0.00
bio_14	1.23	1.27	1.05	0.02	0.01	0.03
bio_16	1.23	1.14	1.08	0.01	0.02	0.00
climaticMoistureIndex	1.18	1.17	1.05	0.02	0.02	0.05
clay	0.86	0.88	0.89	0.01	0.01	0.04
soc	0.68	0.72	0.73	0.05	0.05	0.05

### 3.3 Characterization of the morphological traits in correlation to phylogeny

**FIGURE 2:**
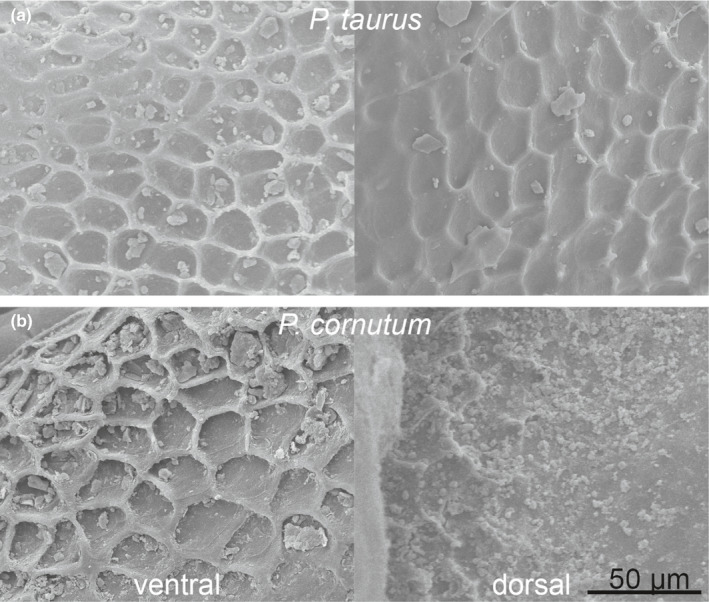
Different manifestation of microstructures on ventral and dorsal scales of two exemplary *Phrynosoma* species. SEM images

According to the phylogenetic signal when analyzing the niche dimensions, we assumed that likewise morphological traits correlate to habitat choice, as morphological traits are likely to be similar in closely related species. We therefore tested the linkage between phylogeny and microstructure characteristics. *Phrynosoma's* Oberhäutchen are covered with pentagonal or hexagonal microstructures (Figure 2). The surface of the scales was not uniformly structured, though, but microstructures were pronounced in the periphery projecting into the channels between the scales. A microstructure was often missing in the middle of the scales, especially on scales of *P*. *cornutum* and on ventral scales of almost all species (to a different degree) except for *P*. *taurus*. On the scales, ~ 1,300 to ~ 2,800 structures per mm^2^ were counted, whereas the mean depth varied from 0 to 7.5 µm (Figure 1, Table S5). On *P. asio* and *P. taurus*, deeper microstructures were characterized (ventral 7/7.5 µm, dorsal 6 µm). Flat structures were detected on the ventral side of *P. modestum* (0 µm) and *P. platyrhinos* (0.5 µm). The most microstructures were counted on *P. taurus* (ventral ~2,800 mm^−2^), followed by *P. modestum* (ventral ~2,700 mm^−2^) and *P. hernandesi* (ventral and dorsal ~2,600 mm^−2^). Fewest structures were counted for the dorsal side of *P*. *cornutum* (~1,300 mm^−2^), followed by *P. cerroense* (ventral ~1,400 mm^−2^). Comparing depth as well as density of structures (ventrally and dorsally), we found no significant phylogenetic signal (Figure 1, Appendix S1). Hence, morphological traits of the Oberhäutchen are not linked to phylogeny of *Phrynosoma*.

### 3.4 Correlation of morphology and habitat

**FIGURE 3:**
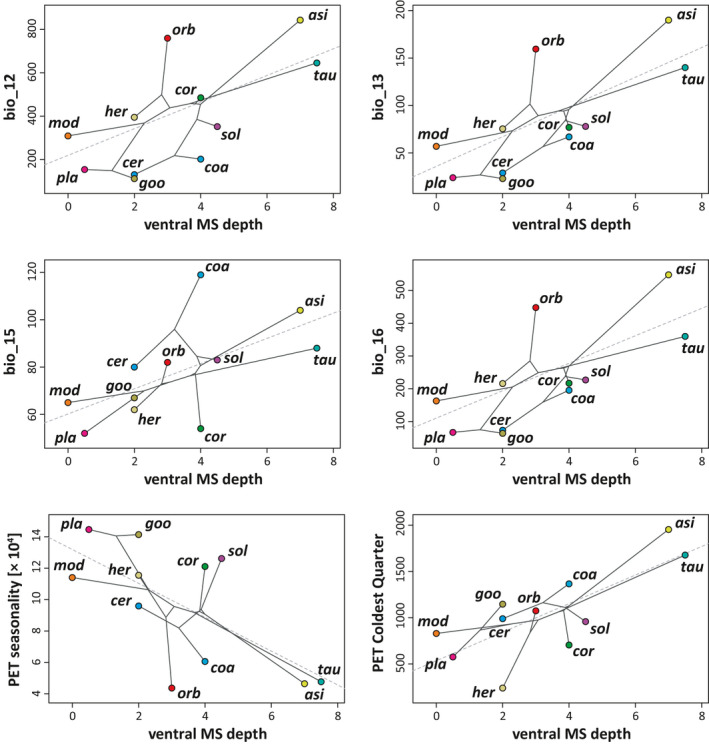
Significant correlations between hydric niche dimensions and ventral microstructure depth as revealed by pgls analyses. None of these correlations remained significant when correcting for alpha error accumulation using false discovery rates. Abbreviations: bio_12 annual mean temperature; bio_13 precipitation of the wettest month; bio_15 precipitation seasonality; bio_16 precipitation of the wettest quarter; PET potential evapotranspiration. Species are abbreviated with the first three letters of their specific epithet

Though morphological traits are independent of phylogeny, habitat choice was linked to morphological traits additionally to being linked to phylogenetic relations. By testing if morphological features can be explained by climatic factors, the phylogenetic generalized least squares regressions suggested a significant correlation in 9 of 175 tests when treating tests separately (Table 3, Appendix S1, but see potential caveats below). Annual mean precipitation (bio_12), precipitation of the wettest month as well as quarter (bio_13, bio_16), precipitation seasonality (bio_15), potential evapotranspiration (PET) of the coldest quarter, and PET seasonality were significant predictors of ventral microstructure depth (six cases, Figure 3) and the interaction of ventral and dorsal microstructure depth (two cases). Only one pedological parameter was significantly correlated with morphology (silt ~number of dorsal microstructures).

**TABLE 3:** Summary statistics of significant phylogenetic generalized least‐square regressions. P‐values were adjusted using the FDR method of p.adjust in R. Significance is marked in red. For abbreviations, see Table 1. MS: microstructure

Correlation	Estimate	*SE*	*t* Value	Pr(>|*t*|)	*R* ^2^	Adj. *R* ^2^	*F*‐statistic	*df*	*df*	*p*	Adj. *p*
PETColdestQuarter ~ ventral MSs depth + dorsal MSs depth											
(Intercept)	185.30	381.30	0.49	0.64	.63	.53	6.73	2	8	.019	.269
Ventral MSs depth	125.16	52.19	2.40	0.04							
Dorsal MSs depth	102.34	98.92	1.03	0.33							
bio_15 ~ ventral MSs depth + dorsal MSs depth											
(Intercept)	35.43	19.25	1.84	0.10	.57	.46	5.27	2	8	.035	.269
Ventral MSs depth	4.88	2.65	1.84	0.10							
Dorsal MSs depth	5.65	4.34	1.30	0.23							
PETColdestQuarter ~ ventral MSSs depth											
(Intercept)	534.78	177.53	3.01	0.01	.58	.53	12.30	1	9	.007	.147
Ventral MSs depth	154.42	44.04	3.51	0.01							
PETseasonality ~ ventral MSs depth											
(Intercept)	131,912.26	16,557.94	7.97	0.00	.43	.37	6.90	1	9	.028	.147
Ventral MSs depth	−10788.23	4,107.52	−2.63	0.03							
bio_12 ~ ventral MSs depth											
(Intercept)	219.31	141.77	1.55	0.16	.37	.30	5.36	1	9	.046	.172
Ventral MSs depth	61.39	26.52	2.32	0.05							
bio_13 ~ ventral MSs depth											
(Intercept)	35.89	29.15	1.23	0.25	.48	.42	8.31	1	9	.018	.147
Ventral MSs depth	15.64	5.43	2.88	0.02							
bio_15 ~ ventral MSs depth											
(Intercept)	60.16	11.61	5.18	0.00	.37	.30	5.20	1	9	.049	.172
Ventral MSs depth	5.33	2.34	2.28	0.05							
bio_16 ~ ventral MSs depth											
(Intercept)	110.35	85.33	1.29	0.23	.44	.38	7.10	1	9	.026	.147
Ventral MSs depth	41.90	15.73	2.66	0.03							
Silt ~ number of dorsal MSs											
(Intercept)	413.52	61.73	6.70	0.00	.49	.44	8.81	1	9	.016	.336
Number of dorsal MSs	−0.08	0.03	−2.97	0.02							

As our data analyses were explorative, we need to acknowledge the potential of alpha error accumulation, and indeed, accounting for false discovery rates (fdr), none of our correlations remained significant. Testing 175 correlations at an alpha of 0.05 results in an adjusted significance level of 0.00028, which is very unlikely to be met by any correlation considering our sample size and natural variation in both morphological and ecological space. Therefore, our results need to be interpreted with caution.

### 3.5 Comparisons of hydric niches among taxa

**FIGURE 4:**
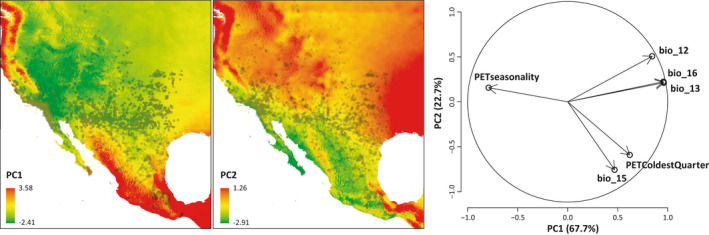
Principal components (PC) and factor loadings based on significant correlations as revealed by pgls regressions. Abbreviations: bio_12 annual mean temperature; bio_13 precipitation of the wettest month; bio_15 precipitation seasonality; bio_16 precipitation of the wettest quarter; PET potential evapotranspiration

A principal components (PC) analysis based on those habitat variables significantly correlated with morphological features suggested two PCs, which in total represent 84.7% of the total variation. PC1 (63.1%, eigenvalue = 3.8) is strongly negatively correlated with PET seasonality and positively correlated with annual mean precipitation (bio_12), precipitation of the wettest month (bio_13), and quarter (bio_16). PC2 (21.7%, eigenvalue = 1.3) is mainly driven by precipitation seasonality (bio_15) and PET of the coldest quarter (cf. correlation circle in Figure 4).

**FIGURE 5:**
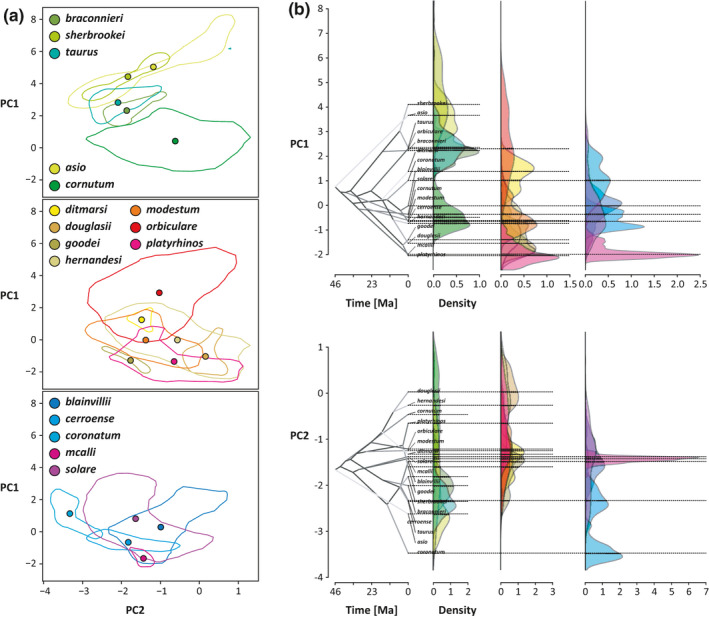
(a) Two‐dimensional hypervolumes of the niches of *Phrynosoma*. Niche space is defined as principal components as shown in Figure 4, wherein subclades are shown separately in the same PCA space to facilitate readability. (b) Phenograms showing the median distribution of *Phrynosoma* taxa in hydric niche space. While the tips of the phylogenies indicate the median position of the species in PC space, the positions of the internal nodes are derived from ancestral character reconstructions using Brownian motion. Density distributions indicating niche occupancy across PC space are derived from all species records and medians are indicated as dashed horizontal lines

Comparing the distribution of species in the environmental space revealed strongly varying degrees of niche position and breadth (Figure 5). The niche space as suggested by the hypervolume analyses revealed that *P. orbiculare* occupies the largest niche space (14.7), followed by *P. cornutum* (10.7), *P. hernandesi* (10.6)*, P. solare* (10.1)*, P. modestum* (6.1)*, P. blainvillii* (6.1)*, P. platyrhinos* (5.7), *P. asio* (5.2)*, P. taurus* (2.4)*, P. douglasii* (1.8)*, P. cerroense* (1.7)*, P. coronatum* (1.6)*, P. sherbrookei* (1.3)*, P. braconnieri* (1.2)*, P. ditmarsi* (0.4)*, P. goodei* (0.4), and *P. mcallii* (0.4). According to our niche volume analysis and ancestral character reconstruction (Figure 5), the realized niche breadth is comparatively smaller and more segregated in PC1 than in PC2.

### 3.6 Wettability of *Phrynosoma*

**FIGURE 6:**
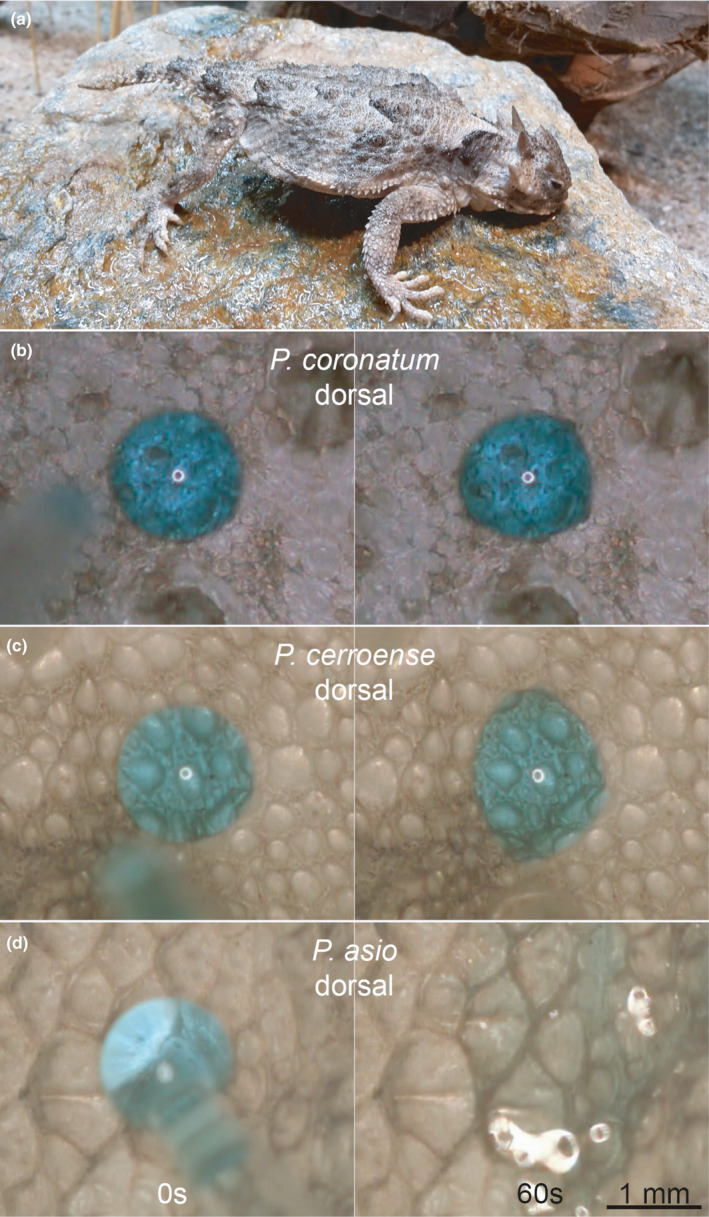
(a) Stereotypic moisture‐harvesting posture exhibited by *Phrynosoma platyrhinos* in captivity. (b–d) Three different wettabilities observed on dorsal shed skin of different *Phrynosoma* species. *Phrynosoma coronatum* (b) was not wettable and the droplet did not change its shape after 60 s. *Phrynosoma asio* (d) was perfectly wettable and direct spreading hampered measurements of droplet size after 60 s. *Phrynosoma cerroense* (c) represents an intermediate wettable *Phrynosoma* species and the droplet spread, but was still visible as droplet after 60 s

Foreshadowing a link between morphology of the skin and habitat choice, we finally tested whether surface structures indeed increase wettability. We detected large differences in wettability of *Phrynosoma* species: Neither all species nor both body sides of one species (dorsal or ventral) were equally well wettable (Figures 1 and 6, Table S6). Only the shed skin of four of the nine tested species (*P. asio*, *P. cerroense*, *P. hernandesi*, and *P. modestum*) showed a prompt spreading of water on their skin (i.e., perfect wetting), and only for *P. modestum,* this was true for both body sides (Figure 6, Table S6). This pattern of wettability could be replicated in live animals (only dorsal side): *P. asio*, *P. modestum*, and *P. solare* (no shed skin samples available for the latter species) proved to be perfectly wettable, whereas water did not spread well on the skin of *P. platyrhinos* and *P. taurus*. The differences in wettability were neither following an obvious phylogenetic nor morphological pattern. We additionally observed that the stereotypic moisture‐harvesting behavior does not have to indicate a good wettability of the skin: Even though *P. platyrhinos* performed the stereotypical behavior (Figure 6a), the wettability of its skin was rather low compared to other *Phrynosoma* species.

### 3.7 Contamination as an influencing factor?

**FIGURE 7:**
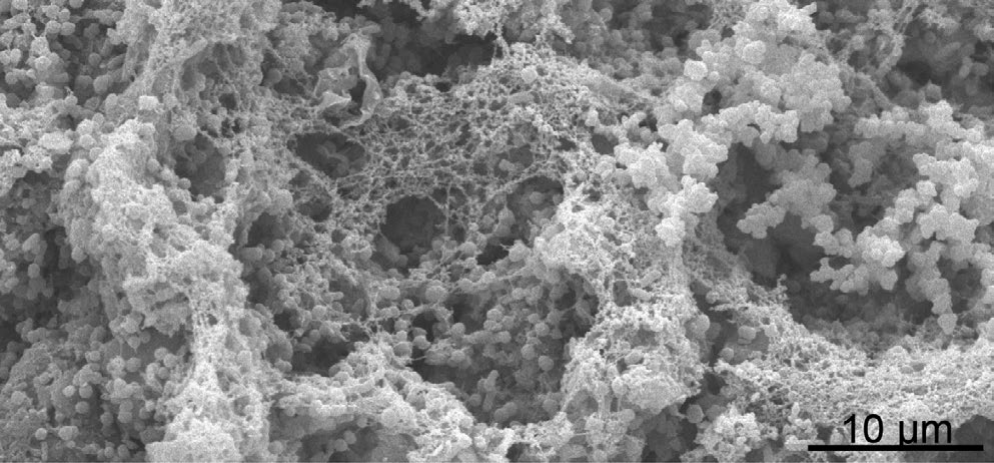
The skin sample of *Phrynosoma solare* with rod‐like and spherical dirt particles. Such particles were only found on *P. solare*. SEM image

The “non‐predictability” of wettability by neither morphological trait nor phylogeny would stay in contrast to the described correlation between some climatic factors and morphological traits. However, applying fdr corrections advise us to interpret some of our data with caution. During SEM analysis, we encountered problems with contaminants on the scales of almost all *Phrynosoma* species, varying in shape and size as well as quantity even within one individual (Figures 2 and 7). None of our cleaning approaches resulted in successful removal of these contaminants. We hypothesized contamination might be a concomitant feature of the microstructure and could be the link between morphology, phylogeny, and hydric and pedological niches.

For a first insight into such an interplay, we analyzed the origin of the contaminations on museum skin samples of *P. cornutum*, for which moisture harvesting has been described in literature, as well as *P. solare*, which has been observed to be perfectly wettable (tests in live animals). Additionally, we included the moisture‐harvesting lizard *Moloch horridus* in our study. *M. horridus* is often used as a comparison species for *Phrynosoma*, as it inhabits a similar habitat in Australia, but developed moisture‐harvesting independently of *Phrynosoma* (Meyers & Herrel, 2005; Pianka & Parker, 1975; Pianka & Pianka, 1970; Sherbrooke, 1990, 1993; Sherbrooke et al., 2007). All three species exhibited a high amount of contamination on their skin.

**FIGURE 8:**
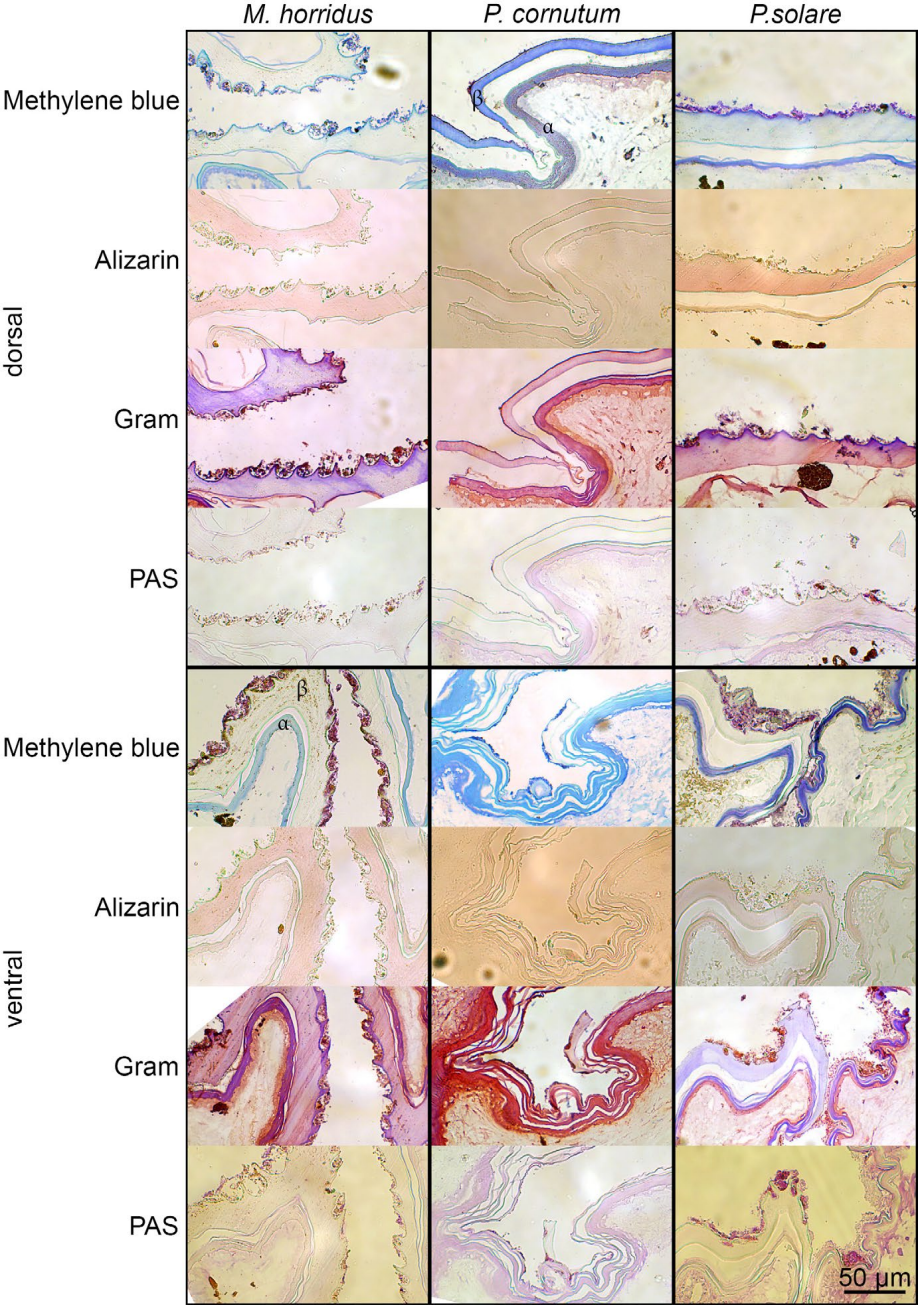
Histological examination of contamination. Semi‐thin sections of scales were stained with either methylene blue, alizarin red, gram staining, or periodic acid‐Schiff staining (PAS). In methylene blue staining, visible skin layers (α‐ and β‐layer) were tagged for the ventral side of *Moloch horridus* with the respective Greek letter for clearer depiction of used sections

Semi‐thin sections of resin‐embedded skin of *P. cornutum*, *P. solare,* and *M. horridus* were stained with methylene blue. We observed the contamination to be stained more strongly than the skin and purple instead of blue (Figure 8). Alizarin red dyes inorganic particles, while, for example, skin layers stay unstained. We observed stained particles within the contamination, often measuring only a micrometer or less in diameter. To check for organic particles, we performed a gram staining and it colored both, skin and contamination. Depending on the presence of α‐ or β‐keratins, skin layers turned red (α‐layer) or purple (β‐layer). The contamination was mainly stained red. A test for collagens and cell walls of fungi via periodic acid‐Schiff staining revealed no coloration in samples of *P. cornutum* nor *M. horridus*, but rod‐shaped and spherical structures on *P. solare*, as also observed in SEM images (Figure 7), were heavily stained pink.

## 4 DISCUSSION

Previous studies linked the superhydrophilic skin of moisture‐harvesting lizards to the occurrence of honeycomb‐shaped microstructures. Our results show a correlation of precipitation in the habitat with the ventral microstructure depth as well as a correlation of hydric as well as the pedological niche dimensions with phylogeny, though morphological traits and phylogeny were independent of each other. However, all other morphological traits as well as the skin's wettability itself could not predict the habitat choice of *Phrynosoma* species. Furthermore, applying fdr corrections to control for potential alpha errors revealed that none of these correlations remained significant. We conclude that it is unlikely that the microstructures influence the wettability, at least directly. We speculate that *Phrynosoma* and other moisture‐harvesting lizards could deploy the soil of their habitat to influence the skin's wettability. Future studies have to validate this hypothesis.

### 4.1 Evolution of environmental niches

Wiens et al. (2013) suggested that *Phrynosoma* originated in comparatively arid environments and mesic habitats were occupied only later. Adaptation to arid environments may therefore be the ancestral condition. According to our dated phylogeny, diversification within the genus mainly happened 15–46 Ma ago (Figure 1) and within this period, climatic conditions in Northern and Central America have been subject to numerous fluctuations and changes (Chapin, 2008). Interestingly, we found a phylogenetic signal for the choice of habitat, regarding precipitation‐related variables and two of three pedological factors. This is partly in concert with previous findings: Analyzing both temperature and precipitation‐related variables, Luxbacher and Knouft (2009) found strong phylogenetic signals only in a single climatic niche axis described by a principal component analysis (PCA) but not in morphological features. This niche axis was related to annual precipitation, precipitation of wettest month, precipitation of wettest quarter, temperature seasonality, maximum temperature of warmest month, and temperature annual range. However, the authors suggested that when accounting for phylogenetic autocorrelation, climatic niche axes were correlated with morphological features (residuals of length measurements, excluding body size) indicating morphological adaptations to environmental conditions. These findings are very similar to what we have found for the microstructures, which may have evolved after speciation as a response to habitat features (i.e., convergent evolution) rather than enabling habitat use in the first place. A comparison between *P. goodei* and *P. cerroense* illustrates this very nicely: They are distantly related and *P. cerroense* inhabits the Baja California Peninsula whereas *P. goodei* lives on the mainland in a similar habitat. This is reflected in very similar ecological niches, for example, regarding precipitation (Figure 3), but also very similar traits of microstructures (aside number of ventral structures). Interestingly enough, both species exhibit different wettability properties, with *P. cerroense* being well wettable and *P. goodei* is not.

In contrast, *P. goodei* and *P. platyrhinos* are closely related species with limited habitat overlap. In accordance with the prediction that phylogeny influences niche choice, their habitats showed, for example, similar annual precipitation (Figure 3). Nevertheless, aside from the dorsal depth of microstructures, both are morphologically distinct and equally poorly wettable. On the contrary, the next related species, *P. modestum*, has no habitat overlap with both and is superhydrophilic. It has equally flat ventral microstructures compared to *P. platyrhinos* and similar quantity of dorsal microstructures compared to *P. goodei*. We found correlations for both these features with hydric (ventral depth), respectively, pedological (dorsal quantity) niche dimensions.

Though we found correlations between morphological traits and niche, applying fdr corrections to control for potential alpha errors erased all correlations. Hence, our data have to be interpreted with caution and we assume it is rather unlikely that the microstructures (directly) influence wettability. Skin structures have been shown to influence tribological properties (Baum et al., 2014), which could be reflected in worn down microstructures at exposed parts of the scales in *Phrynosoma*.

### 4.2 Moisture harvesting in *Phrynosoma* spp

*Phrynosoma* is a typical exemplary genus for moisture (or rain) harvesting lizards, and for three species, the stereotypical behavior has already been described. For *P. modestum*, our study could confirm that moisture harvesting implies a superhydrophilic skin. However, for *P. platyrhinos,* we found the contrary: the stereotypical moisture‐harvesting behavior was accompanied by a badly wettable skin with only few and flat microstructures. Interestingly, *P. hernandesi* was formerly observed to conventionally drink when thirsty (Dodge, 1938), but we characterized their skin as superhydrophilic. Future studies hence should also focus on the important feature of the wettability of the skin when describing moisture harvesting in reptiles.

Four of the five species with the largest niche space characterization show a superhydrophilic skin (*P. cornutum* is described as moisture harvesting having a predominant flow direction of spreading water (Comanns et al., 2015)). Hence, this ability might enable a dispersal to new ecological niches and/or an evolutionary advantage in comparison with other *Phrynosoma*. For example, the habitat of the very distantly related *P. asio* and *P. taurus* largely overlaps, but the realized niche space of the superhydrophilic *P. asio* is larger. However, *P. orbiculare* occupies the largest realized niche space of all *Phrynosoma* and showed no special wettability characteristics. Harvesting water well is not the only selection pressure: *P. cornutum*, with the second largest niche space, is now listed as vulnerable (or worse) species in almost all its habitats in US states (https://explorer.natureserve.org/). The number has declined due to loss of habitat, human eradication of prey ants, invading fire ants, and collection as pet animals (Pianka & Hodges, 2021).

### 4.3 Origin of sample contamination

It was surprising to find an indicated link between deeper ventral microstructures and higher precipitation, as the ventral side of *Phrynosoma* is not exposed to rain. On the other hand, higher precipitation also leads to wetter soil, which has regular contact to the ventral integument. Analyzing the relation between *Phrynosoma's* morphological traits and soil parameters revealed that the occurrence of silt (particle size: ≥0.002 and ≤0.05 mm) in the habitat correlates with the number of dorsally located microstructures. Particles of the contamination on our samples were stained by alizarin red and thus contain calcium (Mulisch & Welsch, 2015). Since most *Phrynosoma*, and also our comparison species *Moloch horridus*, live in desert regions containing limestone and/or sand and sandy loams, stained particles are most likely sand dust, including silt (American Association for the Advancement of Science, 1896; Pianka, 1991; Pianka & Pianka, 1970). There was no phylogenetic signal for silt, but other soil parameters in the habitat (clay and organic particles; Table 2). Hence, we speculate a link between silt and microstructures could have developed independently.

Additionally, there was a strong signal for shed skin, especially remnants of the lower α‐layer. An accumulation of fragments of old skin seems plausible due to the lizards’ innate shedding behavior and these skin remnants would be stained by gram staining due to the amount of cystine in α‐keratin (Alibardi, 1998; Fischer, 1953). The organic contamination on the *P. solare* scales (both sides) also consisted of fungi, seen in the SEM as well as in our histological examinations. Fungi are found on several healthy reptiles as biofilm (Paré et al., 2003). We found fungi only on *P. solare*, and hence, assume this contamination was due to a captured unhealthy animal or incorrect storage of samples and has no functionality.

### 4.4 Influence of the honeycomb‐shaped microstructure on wettability

If the microstructure of moisture‐harvesting lizards’ scales would be the key factor for the efficient spreading of water on their skin, being clean would be essential. However, *Phrynosoma* were never directly observed nor described in literature to perform grooming or similar behavior. On the contrary, *Phrynosoma* as well as *M. horridus* evolved habits that increase their level of contamination: Depending on the species, behaviors like rubbing their venters on the ground, shoveling sand on their dorsal surface, or burrowing themselves in a sandy and dusty substrate were described (Comanns et al., 2016; Pianka et al., 1998; Sherbrooke, 1993; Weese, 1919). All of these can contribute to silt accumulating in the honeycomb‐shaped microstructures of the lizards’ scales. Additionally, the lizards’ skin often sheds in pieces, especially the α‐layer in direct contact with the new forming Oberhäutchen (Maderson et al., 1998). These residual skin pieces complement the contamination. Therefore, we hypothesize that the scales’ microstructure does not contribute to the lizards’ wettability directly, but can serve as container for contamination. Smaller particles, like shed skin and especially silt, would influence the wettability positively due to (a) smaller capillaries forming between the grains and particles and (b) in case of silt: reduced contact angle compared to the skin's keratin due to different material chemistry. Further investigations should evaluate this by, for example, comparing the wettability of freshly shed animals (no silt accumulation) to animals exposed to silt in a controlled manner.

## CONFLICT OF INTEREST

The authors declare no conflict of interest.

## AUTHOR CONTRIBUTION

**Anna‐Christin Joel:** Conceptualization (lead); Data curation (equal); Investigation (equal); Supervision (equal); Visualization (equal); Writing‐original draft (lead). **Jenice R. N. Linde:** Data curation (equal); Investigation (equal); Writing‐original draft (supporting). **Philipp Comanns:** Conceptualization (equal); Funding acquisition (lead); Project administration (equal); Supervision (equal); Writing‐review & editing (supporting). **Caroline Emonts:** Data curation (equal); Investigation (equal); Writing‐review & editing (supporting). **Margret Weissbach:** Data curation (equal); Investigation (equal); Writing‐review & editing (supporting). **Morris Flecks:** Data curation (equal); Formal analysis (equal); Visualization (equal); Writing‐review & editing (equal). **Dennis Rödder:** Conceptualization (equal); Data curation (equal); Formal analysis (equal); Investigation (equal); Validation (equal); Visualization (equal); Writing‐original draft (equal).

## Supporting information

Table S1‐S7

Supplementary Material

Supplementary Material

Supplementary Material

## Data Availability

The supplement includes the following tables: S1 Samples used for investigation. S2 Taxon sampling and GenBank accession numbers. S3 Used loci and dataset properties. S4 Estimated divergence times. S5 Mean morphological traits. S6 Mean wettability of shed skin of *Phrynosoma*. S7 Raw data. Additionally, Appendix S1 includes the detailed statistical tables. Our phylogenetic data are also provided as nexus and newick file.
