# Supplementary material for: Phylogenetic and morphological influence on habitat choice in moisture‐harvesting horned lizards (Phrynosoma spp.)
Source: Ecol Evol. 2021 Sep 21;11(20):14146–61. doi: 10.1002/ece3.8132 (PMC8525137; doi:10.1002/ece3.8132)
Supplement: Supplementary file 1 — Table S1‐S7 [file ECE3-11-14146-s001.pdf]

Supplement

Phylogenetic and morphological influence on habitat choice in moisture harvesting horned lizards (*Phrynosoma* spp.)

Anna-Christin Joel[1+*], Jenice R. N. Linde[1+], Philipp Comanns[1], Caroline Emonts[1], Margret Weissbach[1], Morris Flecks[2], Dennis Rödder[2*]

1: RWTH Aachen University, Institute of Biology II, Worringerweg 3, 52064 Aachen, Germany

2: Zoologisches Forschungsmuseum Alexander Koenig, Adenauerallee 160, 53113 Bonn, Germany

+: These authors contributed equally

Corresponding authors (*):

Anna-Christin Joel

RWTH Aachen University, Institute of Biology II

Worringerweg 3, 52074 Aachen, Germany

joel@bio2.rwth-aachen.de; +49-241-8026554

Dennis Rödder

Zoologisches Forschungsmuseum Alexander Koenig

Adenauerallee 160, 53113 Bonn, Germany

d.roedder@leibniz-zfmk.de; +49-228-9122252

Table S1. Samples used for investigation.

Light blue (+): shed skin, blue (#): museum specimen, dark blue (*): alive animals, grey: data from a database, hatched/two symbols: samples from two different sources were used.

Species	Specimen source			Analysis			
	shed skin	museum	living	Wetting	Morphology	Histology	Phylogeny
Phrynosoma asio	+	#	*	+ *	+ #		
P. cerroense	+	#		+	+ #		
P. cornutum		#			#	#	
P. coronatum	+			+	+		
P. goodei	+			+	+		
P. hernandesi	+	#		+	+ #		
P. modestum	+		*	+ *	+		
P. orbiculare	+	#		+	+ #		
P. platyrhinos	+	#	*	+ *	+ #		
P. solare		#	*	*	#	#	
P. taurus	+	#	*	+ *	+ #		
P. blainvilli							
P. braconnieri							
P. ditmarsi							
P. douglasii							
P. mcallii							
P. sherbrookei							
Moloch horridus		#				#	

Table S2. Taxon sampling and GenBank accession numbers.

Species	BDNF	EXPH5	NKTR	R35	RAG1	SOCS5
Phrynosoma asio	KJ124058	KJ124027	KJ124093	KJ123997	KJ123981	KJ123951
Phrynosoma blainvillii	DQ385337	KJ124030	KU745033	KU745153	DQ385425	-
Phrynosoma braconnieri	KJ124062	KJ124034	KJ124099	KJ124003	KJ123985	KJ123957
Phrynosoma cerroense	DQ385338	KJ124035	KJ124100	KJ124004	DQ385426	KJ123958
Phrynosoma cornutum	DQ385323	KJ124036	KJ124101	KJ124005	DQ385411	KJ123959
Phrynosoma coronatum	DQ385335	KJ124037	KJ124102	KJ124006	DQ385423	KJ123960
Phrynosoma ditmarsi	DQ385333	KJ124038	KJ124103	KJ124007	DQ385421	KJ123961
Phrynosoma douglasii	KJ124063	KJ124039	KJ124104	KJ124008	KJ123986	KJ123962
Phrynosoma goodei	DQ385331	KJ124040	KJ124105	KJ124009	DQ385420	KJ123963
Phrynosoma hernandesi	DQ385324	KJ124041	KJ124106	KJ124010	DQ385412	KJ123964
Phrynosoma mcallii	DQ385328	KJ124042	KJ124107	KJ124011	DQ385417	KJ123965
Phrynosoma modestum	DQ385325	KJ124043	KJ124108	KJ124012	DQ385413	KJ123966
Phrynosoma orbiculare	KJ124064	KJ124044	KJ124109	KJ124013	KJ123987	KJ123967
Phrynosoma platyrhinos	DQ385330	KJ124045	KJ124110	KJ124014	DQ385419	KJ123968
Phrynosoma sherbrookei	KJ124071	KJ124054	KJ124119	KJ124023	KJ123993	KJ123977
Phrynosoma solare	DQ385327	KJ124046	KJ124111	KJ124015	DQ385415	KJ123969
Phrynosoma taurus	KJ124072	KJ124055	KJ124120	KJ124024	KJ123994	KJ123978
Sceloporus bicanthalis	KF422148	KF422193	KF422330	KF422460	KF422505	KF422549

Table S3. Used loci and dataset properties.

Locus	Taxa	Length [bp]	Seg. sites	Inf. sites	Subst. model
BDNF	18	670	179	158	GTR+I
EXPH5	18	609	141	18	HKY+G
NKTR	18	638	251	164	HKY+G
R35	18	704	235	102	HKY+G
RAG1	18	1054	399	244	GTR+I
SOCS5	17	374	30	7	HKY+I

Table S4. Estimated divergence times. Node numbers refer to Figure 1, except for node 0 (stem of *Phrynosoma*). Nodes with an asterisk are calibration points.

Node	Mean height	95 % HPD lower	95 % HPD upper
0*	148.99	91.63	220.24
1	45.93	33.30	64.00
2	42.64	29.75	60.45
3	39.36	27.36	55.82
4	36.24	25.26	51.64
5	34.74	22.23	50.33
6	32.37	22.04	46.14
7*	31.19	19.56	45.83
8	26.42	17.04	38.47
9	23.39	16.16	32.63
10*	16.46	13.6	22.07
11	16.20	8.71	24.94
12	13.29	6.11	21.49
13	9.33	3.96	14.86
14	5.21	1.80	9.10
15	4.77	1.43	8.82
16	3.33	0.87	6.28

Table S5. Mean morphological traits.

	structures mm^{-2}		depth µm^{-1}	
**Species**	**ventral**	**dorsal**	**ventral**	**dorsal**
Phrynosoma asio	2260	2398	7	6
P. cerroense	1391.5	2356	2	3.5
P. cornutum	1917.5	1306	4	4
P. coronatum	1904	1972.5	4	6
P. goodei	2149	2443	2	4
P. hernandesi	2637.5	2553	2	4
P. modestum	2712	2430.5	0	5
P. orbiculare	1620	2234	3	2
P. platyrhinos	1637.5	1668	0.5	3.5
P. solare	1979.5	2223	4.5	4
P. taurus	2841	2377	7.5	6

Table S6. Mean wettability of shed skin of *Phrynosoma*.

Phrynosoma	*asio*	*cerroense*	*coronatum*	*goodei*	*hernandesi*	*modestum*	*orbiculare*	*platyrhinos*	*taurus*
Dorsal	>214%*	93%	-9%	28%	>214%	>214%*	34%	32%*	40%*
Ventral	55%	158%	36%	34%	69%	>214%	49%	19%	101%

*: corroborated on alive animals

Table S7. Raw data. If there are < 3 data points for one animal, the microstructure was either too flat or dirty for sensible measurements.

MS: microstructure. S: Shed skin sample. M: museum specimens. >>: very fast spreading

P. asio

	Number of MSs [mm^{-2}]	Depth [µm]	Spreading [%]
ventral:	S: 2260; 2738; 2399<br>S: 2090; 1629; 1785<br>M: 2307; 2166; 2569	S: 5; 8; 9; 5; 4; 4<br>M: 7; 7; 6; 14; 13; 11; 11; 9; 12<br>M: 3; 3; 7; 3; 5; 5	S: 22<br>S: 64<br>S: 79
dorsal:	S: 2398; 2567; 3289<br>S: 2307; 2812; 2257<br>M: 1965; 2820; 1808	S: 4; 5; 6; 5; 7<br>M: 9; 7; 6; 11<br>M: 5; 6; 6	S: >><br>S: >><br>S: >>

P. cerroense

	Number of MSs [mm^{-2}]	Depth [µm]	Spreading [%]
ventral:	S: 1258; 846<br>S: 1337; 927; 2362<br>S: 1538; 1446; 1971	S: 6; 6; 13; 0<br>M: 0; 3; 6; 4; 4; 0; 0<br>M: 0; 1; 2; 2; 2; 0; 3; 0	S: >><br>S: >><br>S: 47
dorsal:	S: 2761; 2271; 2356<br>S: 2867; 2117; 2933<br>S: 1617; 2411; 1712	S: 8; 4; 7<br>M: 7; 0; 3; 1; 3; 3<br>S: 5; 6; 0	S: 20<br>S: 44<br>S: >>

P. cornutum

	Number of MSs [mm^{-2}]	Depth [µm]	Spreading [%]
ventral:	M: 1697; 2163; 2172<br>M: 1990; 1791<br>M: 1714; 2026; 1845	M: 5; 4; 2; 2; 3; 8; 8; 7; 5; 3; 7; 4; 2; 2; 2; 3; 4<br>M: 6; 9; 7; 4; 8; 9; 4; 4; 4<br>M: 0	
dorsal:	M: 1270; 1342<br>M: 986; 1037; 580<br>M: 2306; 1854; 2013	M: 2; 2; 5; 6; 3<br>M: 15; 1; 2; 7; 5; 8<br>M: 4; 5; 2; 3; 5; 5; 4; 5; 4; 4	

P. coronatum

	Number of MSs [mm^{-2}]	Depth [µm]	Spreading [%]
ventral:	S: 1625; 1525; 1654<br>S: 1989; 1854; 1997	S: 4; 3; 7; 6; 7; 4; 4; 6<br>S: 3; 4; 4; 4; 4; 3	S: 15<br>S: 35<br>S: 57
dorsal:	S: 1845; 3783; 1668<br>S: 1942; 2126; 2003	S: 7; 4; 6; 3; 2; 2; 2; 12; 13; 18; 7; 6; 9; 8; 8<br>S: 4; 3; 5; 6; 5	S: -2<br>S: -26<br>S: 0

P. goodei

	Number of MSs [mm^{-2}]	Depth [µm]	Spreading [%]
ventral:	S: 1796; 2413; 1527<br>S: 2043; 2149; 2437<br>S: 2287; 2039; 2195	S: 0; 6; 3; 2; 6; 2<br>S: 2; 2; 4; 1; 1; 2; 1; 2; 1; 0; 2; 1<br>S: 5; 2; 3; 3; 3	S: 16<br>S: 24<br>S: 63
dorsal:	S: 2055; 1911; 1889<br>S: 2936; 3272; 2091<br>S: 2869; 2773; 2443	S: 3; 3; 4; 4; 5<br>S: 8; 6; 10<br>S: 0	S: 10<br>S: 24<br>S: 49

P. hernandesi

	Number of MSs [mm^{-2}]	Depth [µm]	Spreading [%]
ventral:	S: 2534; 2849<br>S: 2941; 2721<br>S: 2243; 2554	S: 2; 2; 2<br>S: 0; 2; 1<br>M: 0	S: 53<br>S: 70<br>S: 85

dorsal:	S: 2556; 2231; 2553<br>S: 2761; 2058<br>S: 2761; 2058	S: 2; 4; 3; 6; 5; 5; 3; 1; 6<br>S: 11; 6; 7; 3; 2; 9; 3; 5; 4; 6; 3<br>M: 3; 2; 3; 3; 3; 2; 6; 3; 6; 4; 4; 4; 4; 4; 5; 5; 5; 5; 9	S: >><br>S: >><br>S: >>

P. modestum

	Number of MSs [mm^{-2}]	Depth [µm]	Spreading [%]
ventral:	S: 2515; 2785; 2439<br>S: 3793; 3379; 2566	S: 0<br>S: 0; 0; 0; 5	S: >><br>S: >><br>S: >>
dorsal:	S: 2849; 3323; 2330<br>S: 2250; 2531; 1901	S: 6; 0; 0; 5<br>S: 4; 3; 4; 6; 6; 7; 9; 7; 5; 8; 6; 5; 4	S: >><br>S: >><br>S: >>

P. orbiculare

	Number of MSs [mm^{-2}]	Depth [µm]	Spreading [%]
ventral:	S: 2997; 2791; 2065<br>M: 1464; 1336; 1664<br>M: 1191; 1408; 1620	S: 3; 3; 5; 4; 7<br>S: 0; 3; 4; 3; 3<br>M: 3; 3; 0; 0; 0	S: 29<br>S: 91<br>S: 28
dorsal:	S: 2157; 2351; 2234<br>S: 1877; 2027; 2307<br>M: 2542; 3071; 2068	S: 2; 3; 4; 4; 6<br>S: 3; 2; 5; 5; 2; 2; 1; 1; 0; 1; 2<br>M: 4	S: 19<br>S: 51<br>S: 31

P. platyrhinos

	Number of MSs [mm^{-2}]	Depth [µm]	Spreading [%]
ventral:	M: 1497; 1865; 1263<br>S: 2482; 1540; 1391<br>S: 1735; 2698	S: 3; 1; 1; 1; 0; 0<br>S: 0<br>S: 0	S: 4<br>S: 6<br>S: 48
dorsal:	S: 1751; 1295; 1668<br>S: 2077; 1646; 1664<br>S: 1804; 2002; 1665	S: 3; 3; 2; 1; 1; 6<br>S: 2; 2; 2; 3; 5; 6; 2; 9<br>S: 5; 4; 5; 3; 3; 11; 8; 3; 7; 4; 6; 5; 3; 3; 4; 7; 2; 4	S: 21<br>S: 42<br>S: 33

P. solare

	Number of MSs [mm^{-2}]	Depth [µm]	Spreading [%]
ventral:	M: 2326; 1311; 1581<br>M: 1775; 2184; 2901	M: 4; 2; 1; 2; 2; 3; 4; 4; 5; 5; 8; 5; 4; 7; 9; 3; 5, 3; 5<br>M: 8; 5; 8	
dorsal:	M: 4; 2; 1; 2; 2; 3; 4; 4; 5; 5; 8; 5; 4; 7; 9; 3; 5; 3; 5<br>M: 8; 5; 8	M: 0; 1; 1; 3; 6; 7; 3; 3; 5<br>M: 10; 9; 6; 4; 4; 4; 3	

P. taurus

	Number of MSs [mm^{-2}]	Depth [µm]	Spreading [%]
ventral:	S: 2841; 3228; 2518<br>S: 3196; 3127; 3084<br>M: 2312; 2163; 2324	S: 5; 7; 13; 0; 4; 4; 3; 3; 3<br>S: 3; 5; 5; 6; 14; 12; 9; 8; 9<br>M: 11; 11; 11; 10; 7; 12; 11; 11; 15; 4; 6; 6; 5; 6; 7; 17; 9; 6; 11; 4; 7; 9; 9; 5; 9; 8; 9; 7; 9; 8; 9; 6; 4; 11; 8	S: 214<br>S: 10<br>S: 78
dorsal:	S: 2389; 2377; 2717<br>S: 2433; 2761; 3036<br>M: 1765; 1940; 1575	S: 6; 12; 6; 3; 6; 7; 6; 0<br>S: 4; 4; 5; 6; 3<br>M: 5; 3; 6; 5; 14; 9; 6; 12; 14; 9; 5; 8; 7; 5; 14; 11; 5; 15; 6; 7; 8; 0	S: 30<br>S: 89<br>S: 2
